# Tumour Necrosis Factor-Alpha (TNF-α)-Induced Metastatic Phenotype in Colorectal Cancer Epithelial Cells: Mechanistic Support for the Role of MicroRNA-21

**DOI:** 10.3390/cancers15030627

**Published:** 2023-01-19

**Authors:** Aminah G. Alotaibi, Jia V. Li, Nigel J. Gooderham

**Affiliations:** 1 Department of Metabolism, Digestion and Reproduction, Imperial College London, London W12 0NN, UK; 2 National Centre for Genomic Technology, King Abdulaziz City for Science and Technology, KACST, Riyadh 11442, Saudi Arabia

**Keywords:** colon cancer, epigenetic, metastasis, miR-21, TNF-α, tumour microenvironment

## Abstract

**Simple Summary:**

The progression of colorectal cancer is promoted by changes in the genetic makeup of tumour cells giving them potential to leave the site of their origin to seed new metastatic tumours in other tissue; inflammation at the tumour site is a driver of these changes. Tumour necrosis factor-alpha is a pro-inflammatory molecule that is associated with the progression of metastatic cancer. Small biologically active RNAs, microRNAs, target the production of specific proteins and are proposed as agents of tumour change. One agent, microRNA-21, shown to be present in colorectal cancer, is known to target the formation of proteins involved in metastatic changes in cells. We investigated the relationship between TNF-α and microRNA-21 in colorectal cancer cells and show their involvement in promoting cell changes indicative of the metastatic state. In summary, we provide mechanistic support for a role of microRNA-21 in tumour necrosis factor-alpha promotion of cancer cell metastatic change.

**Abstract:**

Colorectal cancer is driven by genetic and epigenetic changes in cells to confer phenotypes that promote metastatic transformation and development. Tumour necrosis factor-alpha (TNF-α), a pro-inflammatory mediator, regulates cellular communication within the tumour microenvironment and is associated with the progression of the metastatic phenotype. Oncogenic miR-21 has been shown to be overexpressed in most solid tumours, including colorectal cancer, and is known to target proteins involved in metastatic transformation. In this study, we investigated the relationship between TNF-α and miR-21 regulation in colorectal cancer epithelial cells (SW480 and HCT116). We observed that TNF-α, at concentrations reported to be present in serum and tumour tissue from colorectal cancer patients, upregulated miR-21 expression in both cell lines. TNF-α treatment also promoted cell migration, downregulation of the expression of E-cadherin, a marker of epithelial to mesenchymal transition, and anti-apoptotic *BCL-2* (a validated target for miR-21). Knockdown of miR-21 had the opposite effect on each of these TNF-a induced phenotypic changes. Additionally, in the SW480 cell line, although TNF-α treatment selectively induced expression of a marker of metastatic progression *VEGF-A*, it failed to affect *MMP2* expression or invasion activity. Our data indicate that exposing colorectal cancer epithelial cells to TNF-α, at concentrations occurring in the serum and tumour microenvironment of colorectal cancer patients, upregulated miR-21 expression and promoted the metastatic phenotype.

## 1. Introduction

Colorectal cancer (CRC), the second leading cause of cancer-related mortality [1], is the third most common cancer in men and second in women [2,3]. Whilst age is a factor of the disease, recently the incidence of CRC has increased in those younger than 50 years old [4]. Despite the fact that early detection and advances in treatment have reduced mortality of CRC [5], survival rates have not significantly improved [4]. Thus, there is a need for deeper understanding of the underlying molecular mechanisms involved in CRC to facilitate the development of new diagnostic and therapeutic strategies. 

Chronic inflammation is a known risk factor for CRC development, progression, metastasis, and resistance to cancer therapy [6,7,8]. We have previously shown that the expression of several inflammatory mediators are upregulated in CRC tissue [9,10,11]. Tumour necrosis factor-alpha (TNF-α) is an inflammatory mediator, one of several present at high levels in solid tumours and the serum of CRC patients [12,13,14] that have been implicated in metastatic development. TNF-α concentrations in excess of 36 pg/mL have been reported in the circulation of patients with CRC [13] and levels of up to 25 pg/mL have been reported in CRC tumour tissues [15]. Metastatic changes range from the migration of primary tumour cells to adjacent normal tissues, to migration and invasion to distant organs to create secondary tumours [16]. The specific mechanisms by which cancerous cells acquire the metastatic phenotype are complex and not fully understood, but involve epithelial to mesenchymal transition (EMT). The loss of cellular E-cadherin expression is considered as a hallmark in the transition of non-invasive tumour to invasive carcinoma and constitutes a fundamental step of the EMT [17,18]. It has been shown that TNF-α stimulates EMT by reducing E-cadherin expression in hepatic cancer cells [19] and melanoma cells [20] and to increase cell motility, migration, and invasion in colonic epithelial cells [21,22]. 

MicroRNAs (miRs) are powerful post-transcriptional mediators of gene-expression, the dysregulation of which has been strongly linked to the development of numerous cancers [23,24,25,26,27]. MiRs can regulate the expression of numerous proteins involved with every stage of tumour development including the enhanced expression of proto-oncogenes, inhibition of tumour suppressor genes, and the expression of key proteins involved with cancer-associated pathways and metastases [23,28,29]. Whilst the mechanisms of miR control of gene expression is primarily intracellular, it is now apparent that cell-to-cell crosstalk can be achieved via secretion, transport, and delivery of protein and miR packaged exosomes [30,31]. Within the tumour microenvironment (TME) cell-to-cell communication via micro-vesicles is recognized to be an important factor in tumour cell development and progression to the metastatic phenotype [32], and miR cargos have been proposed as contributors to these phenotypic changes [31]. Patel et al. [31] determined the expression of multiple miRs in HCT116 and SW480 cells in response to treatment with IL-6 and report that the expression of miRs-21 and -29b were particularly affected. Of these two miRs, miR-21 is known to be oncogenic and strongly associated with tumour progression; it is a potential mediator of metastatic potential [25,27] and its expression is upregulated in many tumours [33,34,35,36,37,38,39,40].

Collectively, these observations implicate inflammatory mediators and miRs as agents of tumour progression. In particular, our previous studies have identified the strong association of inflammatory mediators with the progression of colorectal cancer [9,10,11,26] and the ability of TNF-α to promote DNA damage in colorectal cancer cells [41]. Additionally, co-localisation of TNF-α and miR-21 expression, particularly at the tumour leading edge, of colorectal cancer has been reported [42]. However, the driver of the increased expression of miR-21 and its role in the pathogenesis of CRC are not well understood. 

The role of TNF-α in the development of cancer and the associated elevated miR-21 expression has prompted us to investigate potential mechanisms whereby these mediators influence the progression of the metastatic phenotype in colorectal cancer epithelial cells. The SW480 and HCT116 colon cancer epithelial cells were chosen since they represent different metastatic stages, being isolated from a colorectal cancer patient at Duke’s B stage and from a patient at Duke’s D stage, respectively. Here, we report mechanistic evidence that supports a relationship between the pro-inflammatory cytokine TNF-α and miR-21 in the progression of colorectal cancer epithelial cells towards a metastatic phenotype. 

## 2. Materials and Methods

### 2.1. Cell Culture and Treatment

Human colorectal adenocarcinoma cell lines HCT116 and SW480 were purchased from the American Tissue Culture Collection ATCC (LGC Prochem, Middx, UK). Cells were routinely cultured in RPM1640 medium (GIBCO, Life technologies, Paisley, UK) supplemented with 10% fetal bovine serum (FBS), penicillin (100 units/mL), streptomycin (100 μg/mL), and 2 mM L-glutamine all purchased from GIBCO, Life technologies, Paisley, UK. Cells were cultured at 37 °C in a humidified atmosphere with 5% CO_2_ and incubated for various times (up to 5 days) in the absence or presence of TNF-α (0.01, 0.1, 1, 10, and 100 pg/mL) as indicated in the text and figure legends. In some experiments, an NF-kB inhibitor, bortezomib (BZ, 0.13 mM) (LC laboratories), was included in incubations 4.5 h prior to the TNF-α treatments, as indicated in the text and figure legends [41].

### 2.2. Wound Healing Assay 

Cells (10^5^) were plated in a 24-well plate in 1 mL of RPM1640 medium with 5% dextran-coated charcoal-stripped FBS (GIBCO, Life technologies, Paisley, UK) per well for 72 h until confluent. Cells were then wounded in the monolayer using small sterile 10 µL tips to create a channel (at time 0). Cells were washed with PBS (GIBCO, Life technologies, Paisley, UK) and 1 mL of culture medium supplemented with 5% dextran-coated charcoal-stripped FBS was added. TNF-α treatments (0.01 to 100 pg/mL) were added to cells and incubated for up to 72 h; physiological concentrations of TNF-α in tissue has been reported to be ~10 pg/mL [43] and up to 25 pg/mL in colorectal cancer tissue [15]. The distance between the two sides of the wound (channel) was captured at multiple points (at least 4 determinations per data point) with an inverted optical microscope and analysed by Image J software.

Wound closure at time t was calculated as a fraction of the distance between the two sides of the wound at time zero. For graphical presentation, the fraction was converted to a % of the distance at time zero (%migration).

The percentage migration was calculated as follows:


%migration = (average of wound width at 0 h − average of wound width at t h)/average of wound width at 0 h × 100%.

### 2.3. Transwell Migration and Invasion

For migration assay, a 24-transwell insert system (BD Falcon, Oxford, UK) with 8 μm pore size was used. Each well comprised an upper chamber and a lower chamber separated by the 8 mm pore size membrane; both chambers were immersed in culture media. Cells (3 × 10^4^ per well) were plated in 100 µL of culture medium RPM1640 supplemented with 1% dextran-coated charcoal-stripped FBS (GIBCO, Life technologies, Paisley, UK) in the upper chamber of the insert system containing TNF-α (0 to 100 pg/mL). Culture medium containing 10% FBS (100 μL) was added to each of the lower chambers as a chemoattractant and cells were incubated for 72 h. The number of cells that had migrated through the 8 mm pore membrane to the lower chamber was determined by incubation with alamarBlue reagent (10% final volume) (Invitrogen, Life technologies, Carlsbad, CA, USA) according to manufacturer’s protocol (4 h at 37 °C). Under these conditions, alamarBlue is enzymically converted to fluorescent resorufin by viable cells, which can be measured in a spectrofluorometer. The enzymic formation of resorufin is proportional to cell number. Results are expressed as a percentage compared with vehicle control. 

For the invasion assay, Matrigel invasion inserts (USA, catalogue number: 354480) were used. Each Matrigel insert contained a Matrigel coating on an 8 µm pore size PET membrane. The Matrigel matrix-coated inserts were initially hydrated with warm RPMI medium for 2 h at 37 °C. After hydration, the hydrating media was removed and the insert was placed in each well of a 24-well plate. RPMI culture medium (100 μL) supplemented with 5% dextran-coated charcoal-stripped FBS including TNF-α (0 to 100 pg/mL) was added in the Matrigel chambers, and 100 µL of culture medium with 10% FBS were added to the bottom well to act as a chemo attractant. Each well, therefore, comprised an upper chamber and a lower chamber separated by the Matrigel-coated 8 mm pore size membrane; both chambers were immersed in culture media. The number of cells that had migrated through the membrane to the lower chamber compared to the vehicle control was determined using alamarBlue reagent as described above.

### 2.4. Cell Proliferation 

Cell proliferation was determined using alamarBlue (Invitrogen, Life technologies) according to the manufacturer’s protocol. Cells were cultured in 24-well plates at a density of 5 × 10^4^ cells per well. Healthy viable cells maintain a reducing potential within their cytosol to convert alamarBlue reagent into fluorescent resorufin. AlamarBlue was added at a final concentration of 10% for each well and incubated for 4 h at 37 °C for each treatment time point. The resorufin fluorescent intensity was measured in a FLUOstar plate reader (BMG, Labtech, Ortenberg, Germany) at excitation 560 nm/emission 590 nm. The fluorescence intensity is proportional to the cell number.

### 2.5. RNA and MiRNA Extraction

Total RNA and miRNA were extracted from cells using an RNeasy Micro Kit (QIAGENE, Hilden, Germany) according to the manufacturer’s protocol. Briefly, cell pellets (10^6^ cells) were disrupted with QIAzol Lysis Reagent (700 μL) and homogenised with vortex mixing for 1 min. The homogenate was left at room temperature for 5 min to dissociate the nucleoprotein complexes. Chloroform (140 μL) was added with vigorous shaking for 15 s, left at ambient for 2–3 min, then centrifuged (12,000× *g* for 15 min). The upper colourless aqueous phase was transferred to a collection tube and 1.5 volumes of ethanol (100%) was added with mixing, and then, the whole was transferred to an RNEasy MinElute spin column in a collection tube. The tube (spin column) was centrifuged (8000× *g*, 15 s) at room temperature and the flow through was discarded. Buffer RPE (500 μL) was added onto the RNEasy MinElute spin column and the tube centrifuged (800× *g*, 15 s); the flow through was discarded. Ethanol (80%, 500 μL) was added to the RNEasy MinElute spin column, centrifuged (8000× *g*, 15 s), and the flow through discarded. The RNEasy MinElute spin column was then centrifuged at high speed (5 min) to dry the membrane. RNase-free water (14 μL) was added to the spin column membrane and centrifuged at high speed to elute the RNA (total RNA including miRNA). RNA concentrations were measured by nano-spectrophotometer (Implen, Essex, UK) and the purity of the extracted RNA was assessed using the ratio of absorbance at 260/280 nM and 260/230 nm. Extracted RNA samples were stored at −80 °C until required.

### 2.6. Reverse Transcription and RT-qPCR 

The reverse transcription of messenger RNA and micro-RNA was performed as previously described [41] using superscript II (Invitrogen, life Technologies) and TaqMan micro-RNA reverse transcription kits (Applied biosystems, Thermo Fisher Scientific, Oxford, UK), respectively. Quantitative real time PCR was performed using TaqMan predesigned expression assay for E-cadherin (Hs01023895_m1) selected as a biomarker of EMT transition, *BCL-2* (Hs04986394_s1) a predicted mRNA target of miR-21 obtained using TargetScan version 7.0 [44], *GAPDH* (Hs99999905_m1), *MMP2* (Hs00234422_m1), *VEGF-A* (Hs00900054_m1), *U6* (TM&RT:001973), and *hsa-miR-21-5p* (TM&RT:000397). Fast PCR master mix (TaqMan, applied biosystem, life technologies) was used according to the manufacturer’s protocol. The results of the RT-qPCR were normalized to internal control genes *GAPDH* and *U6*. 

### 2.7. MiRNA Inhibitor Transfection 

miRNA mimics and inhibitors were obtained from MirVana (Thermo Fisher Scientific). Cells were cultured in a 24-well plate at 5 × 10^4^ cell/well and incubated with antibiotics-free RPMI 1640 medium (GIBCO, Life technologies, Paisley, UK) overnight. The medium was then replaced with Opti-MEM medium (400 μL/well) (GIBCO, GRAND Island, NY, USA) and cells were transfected with Lipofectamine RNAiMAX reagent (8 μL) (Invitrogen, Carlsbad, CA, USA) with final miRNA inhibitor concentrations of 20 μΜ. Cells were divided into 4 groups: control group (cells only), Lipofectamine-treated cells (reagents only), miR inhibitor control-treated (scrambled miRNA), and miRNA-21 inhibitor-treated. Transfection efficiency was assessed by FAM™ dye, labelled synthetic miRNA (scrambled), where the fluorescent label enables direct assessment of cellular uptake. The fluorescent intensity was measured in a FLUOstar plate reader (BMG, Labtech) at excitation 494 nm/emission 520 nm (POLARstar Galaxy, BMG Lab Technologies).

### 2.8. Statistical Analysis 

All experiments were performed in triplicate for each single treatment and, unless stated in the text or figures, with three independent biological replicates. Data are reported as a mean and standard error of the mean (SEM) unless otherwise stated. GraphPad Prism software (GraphPad Prism 8, GraphPad Software Inc, La Jolla, CA, USA) was used for statistical analyses. Significant differences between independent means were determined using one-way analysis of variance (ANOVA) followed by a Dunnett’s post-test. *p* values < 0.05 were considered statistically significant.

## 3. Results

### 3.1. TNF-α Promotes Cell Proliferation 

**Figure 1:**
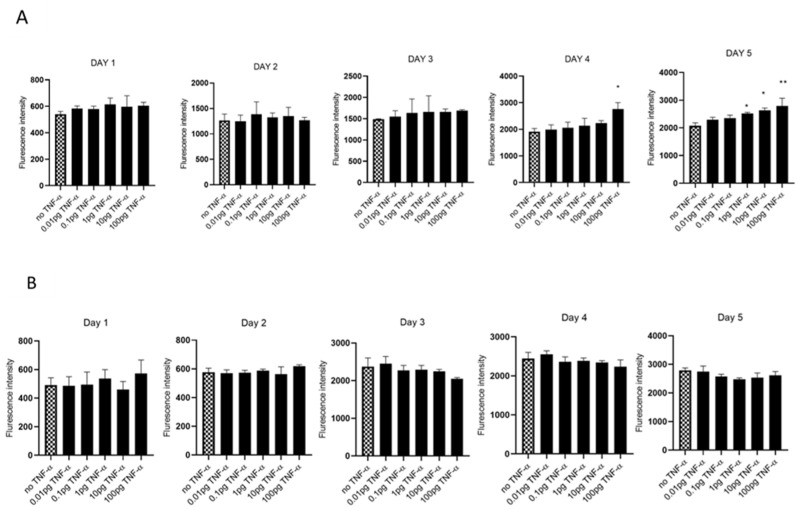
Cell proliferation in response to TNF-α treatment. (**A**) SW480 and (**B**) HCT116 cells were treated with doses of TNF-α (0.01–100 pg/mL) over 5 days. Cell proliferation was determined using alamarBlue assay. Data are expressed as fluorescence intensities compared to vehicle control (no TNF-α treatment). Significant difference was calculated using one-way ANOVA followed by Dunnett’s test (GraphPad Prism 8, ** *p* < 0.01, * *p* < 0.05). Error bars represent the SEM for six replicates and two independent cultures.

We evaluated cell proliferation in HCT116 and SW480 cells treated with physiologically relevant doses of TNF-α (0.01–100 pg/mL) over 5 days, assessed using alamarBlue assay. We found that TNF-α enhanced cell proliferation in SW480 cells at doses of 1, 10, and 100 pg/mL by day 5, but not with HCT116 (Figure 1A,B).

**Figure 2:**
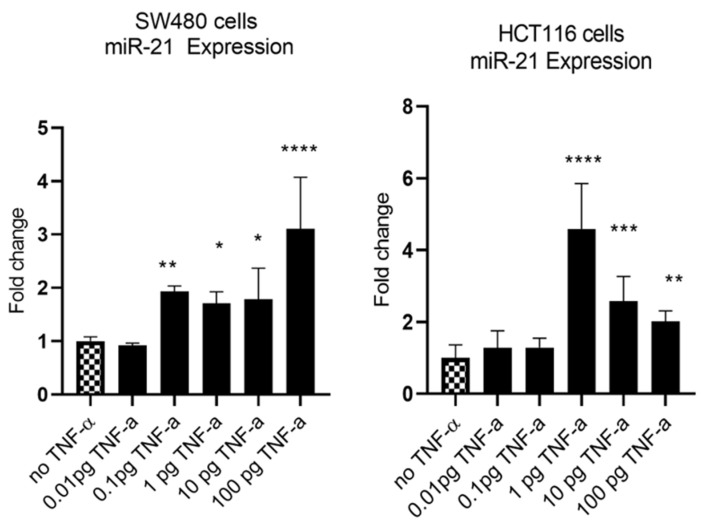
TNF-α effects on miR-21 expression. SW480 and HCT116 cells were treated with 0 to 100 pg/mL of TNF-α for 24 h. MiR-21 expression was measured by RT-qPCR. Data were normalised to expression of U6 RNA and are shown relative to vehicle control (no TNF-α). Significant difference was calculated using one-way ANOVA with Dunnett’s test (GraphPad Prism 8, * *p* < 0.05, ** *p* < 0.01, *** *p* < 0.001, **** *p* < 0.0001). Data are presented as fold changes of three biological replicates relative to control. Error bars represent the SEM for independent cultures.

**Figure 3:**
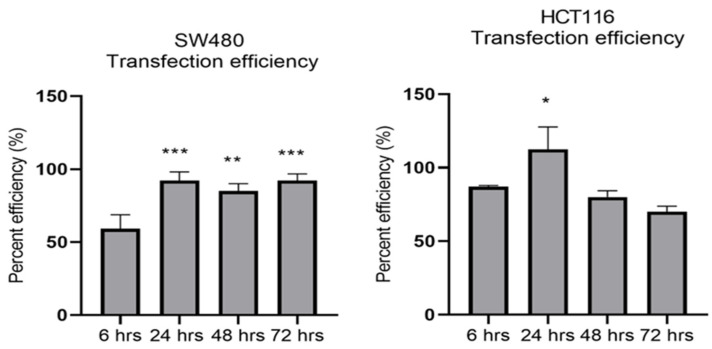
Optimal time for transfection efficiency (TE). FAM dye-labelled synthetic scrambled miRNA control transfected with Lipofectamine RNAiMAX in SW480 and HCT116 cells. Transfected FAM dye-labelled miRNAs was measured by a plate reader at emission 520 nm and excitation 495 nm. Significant difference was calculated using one-way ANOVA with Dunnett’s test comparing to 6 h (the earliest time point) (GraphPad Prism 8, * *p* < 0.05, ** *p* < 0.01, *** *p* < 0.001). Data are presented as a mean percentage of efficiency. Error bars represent the SEM for six replicates and two independent cultures.

**Figure 4:**
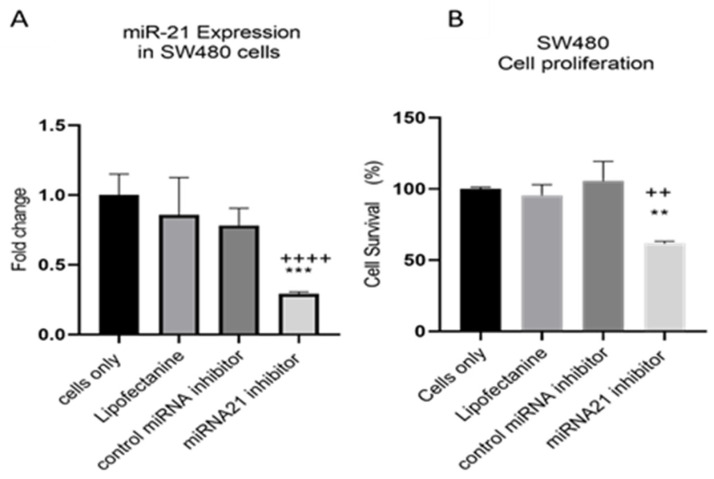
SW480 cells transfected with miR-21 inhibitor. (**A**) miR-21 expression. Data were normalised to expression of U6; (**B**) cell proliferation determined using alamarBlue assay after transfection. Cell proliferation assay was performed over 5 days. Significant differences were calculated using one-way ANOVA followed by Dunnett’s test (GraphPad Prism 8). Data are presented as fold changes or percentages of cell survival of treatment groups relative to untreated group (cells only). Statistical comparisons between cells only vs. miR-21 inhibitor (++++ *p* < 0.001, ++ *p* < 0.01) and control miRNA inhibitor vs. miR-21 inhibitor (** *p* < 0.01, *** *p* < 0.001). Error bars represent the SEM for six replicates and two independent cultures.

MiR-21 has been shown to be upregulated and co-associated with TNF-α expression in colorectal cancer [42] and is positively associated with the progression of a metastatic phenotype [45]. We hypothesised that TNF-α treatment may promote the metastatic phenotype via the expression of miR-21. We, therefore, measured the expression level of miR-21 in response to TNF-α treatment and found it was significantly increased in both cell lines (*p* < 0.05), but with differing dose responses, being elevated at doses ≥ 0.1 pg/mL in SW480 and maximally induced at 1 pg/mL in HCT116 cells (Figure 2). This non-linear dose response is not easy to explain, but optimal effective concentrations of inflammatory mediators invariably peak at physiologically relevant levels, often in the pg/mL range, emphasizing the extreme potency of these biologically active molecules. To evaluate the potential involvement of miR-21 in CRC cell proliferation ability, we transfected mirVana miR-21 inhibitor, to bind and inhibit (knockdown) endogenous miR-21 expression. In preliminary experiments, transfection efficiency was determined by florescence quantification of FAM-labelled miRNA (scrambled) and was optimized for both cell lines at 24 h after transfection (Figure 3). Using a 24-hr transfection protocol, mirVana miR-21 inhibitor significantly reduced miR-21 expression and cell proliferation in SW480 cells (Figure 4A,B).

### 3.2. TNF-a Promotes Cell Migration 

**Figure 5:**
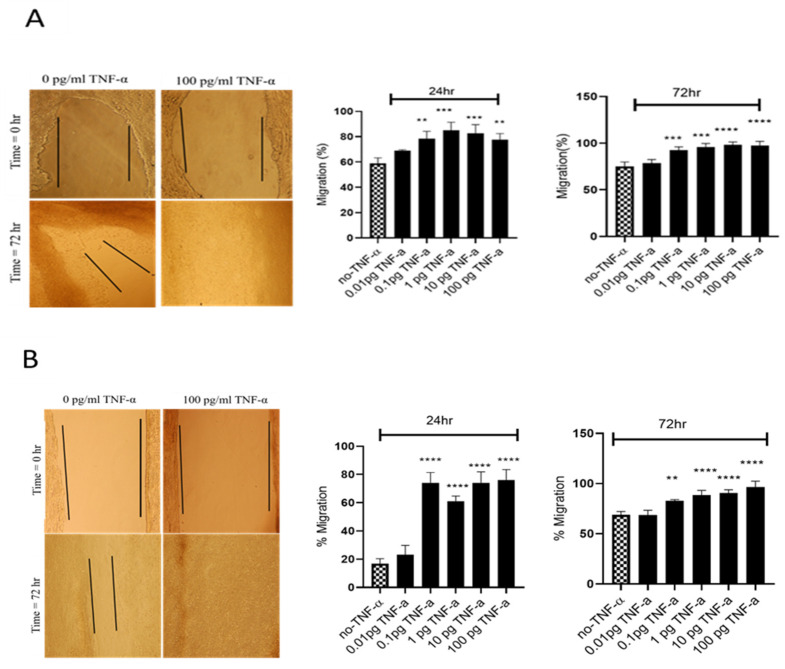
Effects of TNF-α on wound healing in SW480 and HCT116 cells. Wound assays were performed over 72 h using (**A**) HCT116 and (**B**) SW480 cells treated with TNF-α (0.01–100 pg/mL). Pictures of wounded cells taken at 0 and 72 h with or without TNF-α treatment (10× magnification). Data are expressed as % compared to vehicle control (no-TNF-α). Significant difference was calculated using one-way ANOVA with Dunnett’s test comparing treated groups to vehicle control (GraphPad Prism 8, ** *p* < 0.01, *** *p* < 0.001, **** *p* < 0.0001). Error bars represent the SEM for three independent cultures.

To evaluate the proliferation and migratory potential of cells treated with TNF-α, a wound healing assay was performed. In both cell lines TNF-α treatment rapidly closed the wound compared with the controls (*p* < 0.0001) (Figure 5).

**Figure 6:**
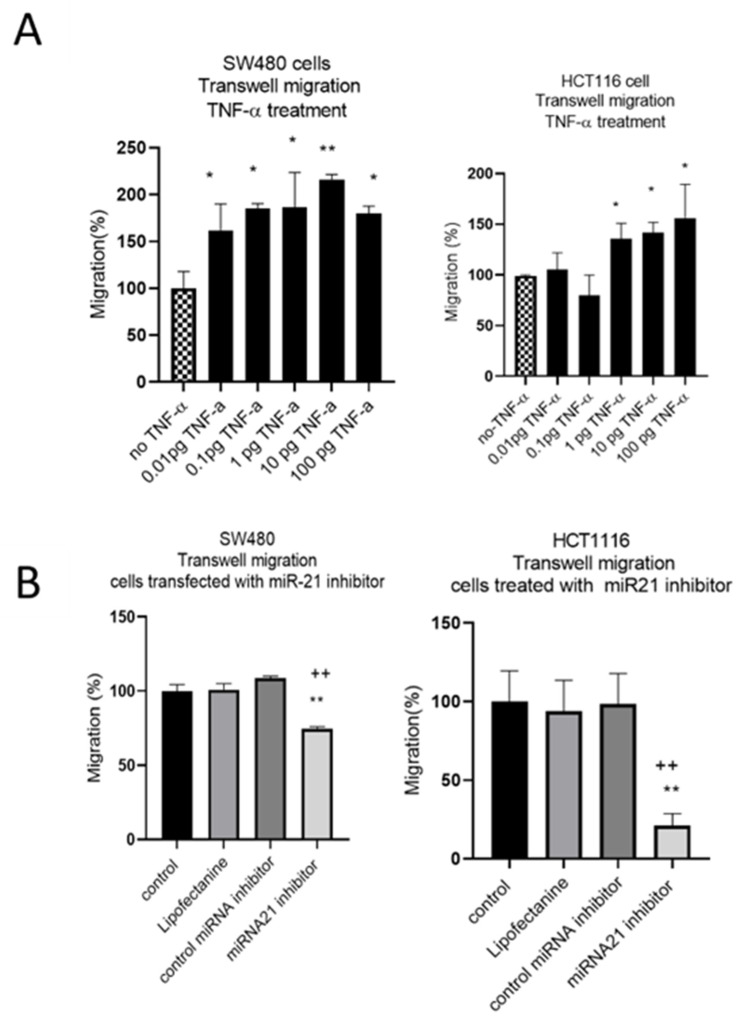
SW480 and HCT116 transwell cell migration (**A**) cells treated with TNF-α. Error bars represent the SEM for three independent cultures; (**B**) cells transfected with miR-21 inhibitor. Error bars represent the SEM for six replicates and two independent cultures. Significant differences were calculated using one-way ANOVA followed by Dunnett’s test (GraphPad Prism 8). Statistical comparisons of TNF-α treatment vs. no TNF-α control, control vs. miR-21 inhibitor (* *p* < 0.05, ** *p* < 0.01), and control miRNA inhibitor vs. miR-21 inhibitor (++ *p* < 0.01).

The results of the wound assay were confirmed with a transwell migration assay that showed a dose-dependent statistically significant increase in cell migration in both cell lines treated with TNF-α (Figure 6A). To investigate whether miR-21 was involved in this induced migratory response, we performed a miR-21 knockdown. In SW480, and particularly in HCT116 cells, miR-21 knockdown resulted in a significant decrease in cell migration (Figure 6B). 

**Figure 7:**
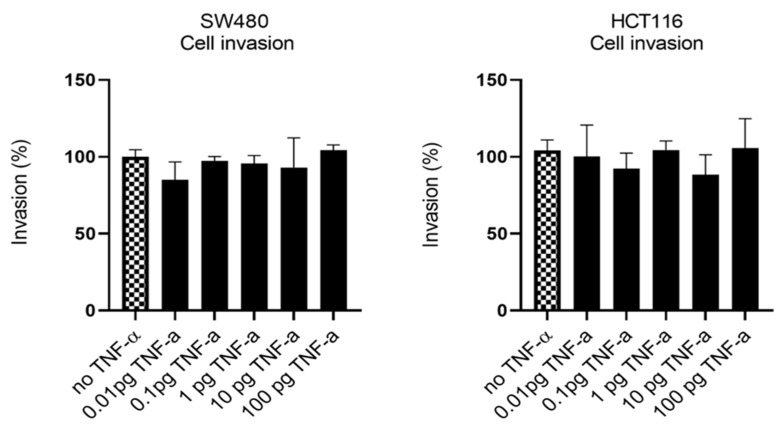
Cell invasion in response to TNF-α treatment in SW480 and HCT116 cells. Cells were treated with a dose range of TNF-α (0.01–100 pg/mL). Cell invasions were determined using transwell Matrigel inserts and alamarBlue assay to assess invading cell numbers. Data are expressed as (%) compared to vehicle control (no TNF-α). Significant difference was calculated using one-way ANOVA followed by Dunnett’s test (GraphPad Prism 8). Error bars represent the SEM for three independent cultures.

Having established that TNF-α promoted migration in SW480 and HCT116 cells, we examined the ability of TNF-α treatment to promote an invasive phenotype using a transwell invasion assay. Irrespective of the TNF-α dose employed, the invasive phenotype was not observed in either cell type (Figure 7).

### 3.3. TNF-α Alters E-Cadherin Expression 

**Figure 8:**
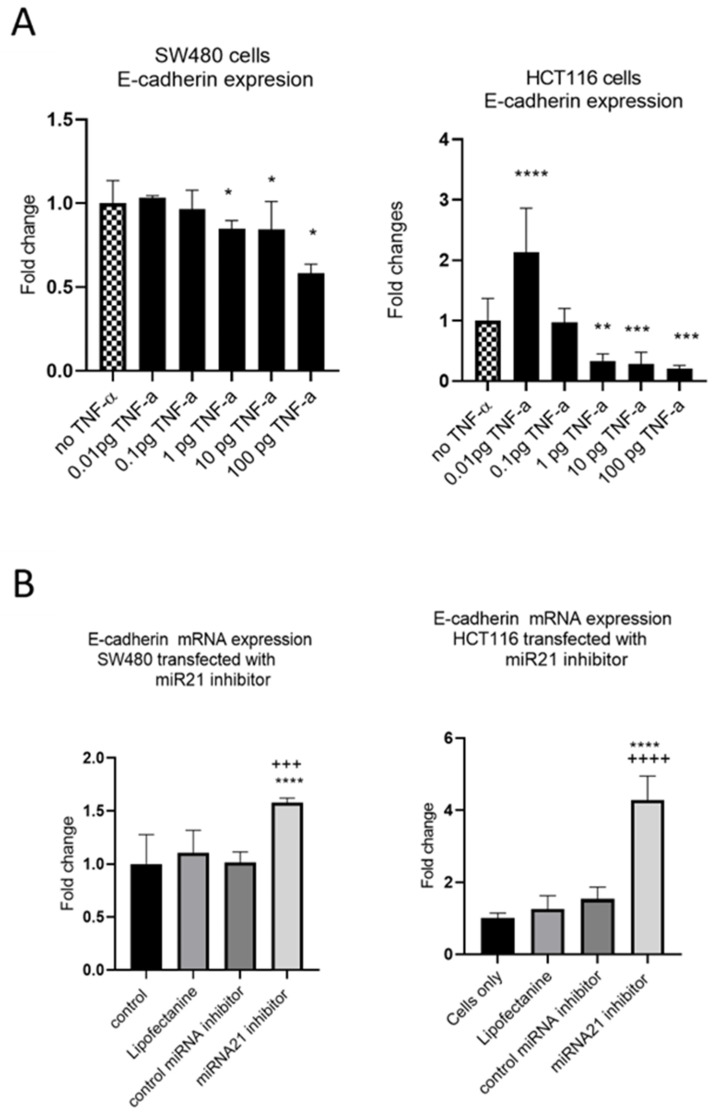
E-cadherin mRNA expression in SW480 and HCT116. (**A**) E-cadherin expression in cells treated with TNF-α. Error bars represent the SEM for three independent cultures; Statistical comparisons of TNF-α treatment vs. no TNF-α control (* *p* < 0.05, ** *p* < 0.01, *** *p* < 0.001, **** *p* <0.0001). (**B**) E-cadherin expression in cells transfected with miR-21 inhibitor. E-cadherin expression was assessed by RT-qPCR. Error bars represent the SEM for six replicates and two independent cultures. Statistical comparisons of control vs. miR-21 inhibitor (**** *p* < 0.0001), and control miRNA inhibitor vs. miR-21 inhibitor (+++ *p* < 0.001, ++++ *p* <0.0001). Significant differences were calculated using one-way ANOVA followed by a Dunnett’s post-test (GraphPad Prism 8).

EMT is an evolutionary program that confers metastatic properties to cells through transformation from the nonmotile epithelial state to a motile mesenchymal state. Thus, we assessed the effect of TNF-α treatment and miR-21 expression on the loss of the epithelial biomarker E-cadherin. In both SW480 and HCT116 cells, treatment with TNF-α (≥1 pg/mL) reduced the expression of E-cadherin (Figure 8A), consistent with the acquisition of a more mesenchymal phenotype. Interestingly, at the very low dose of TNF-α (0.01 pg/mL) in HCT116 cells, E-cadherin, was significantly induced. Furthermore, knockdown of miR-21 resulted in a significant elevation of E-cadherin in both cell types (Figure 8B). 

### 3.4. TNF-a Downregulates BCL-2 Expression 

**Figure 9:**
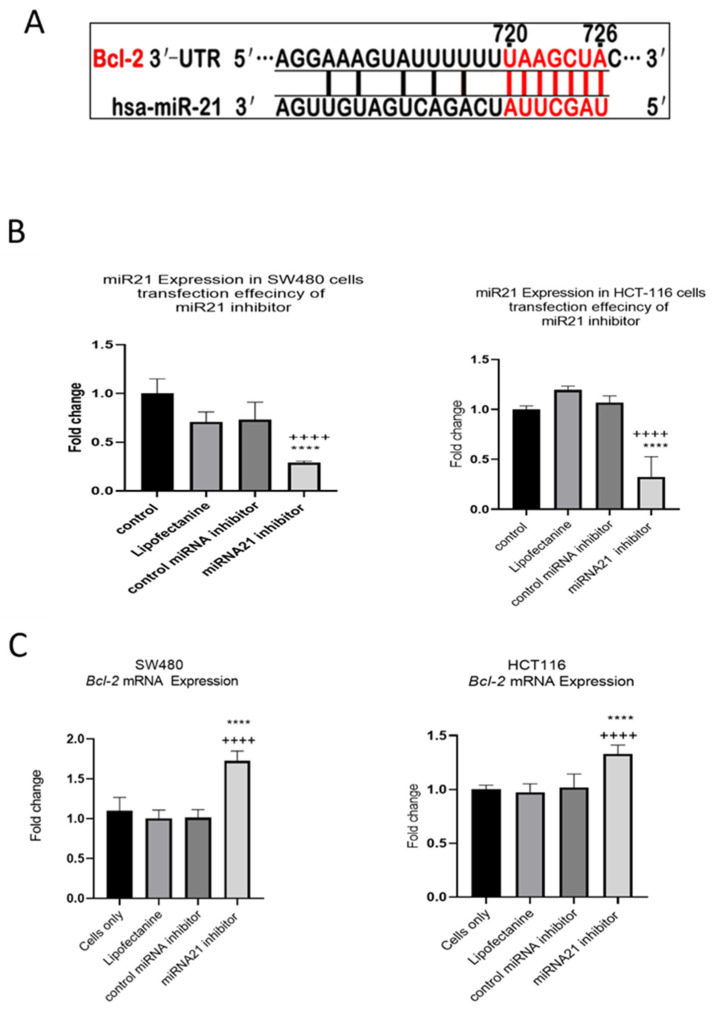
Expression of miR-21 target gene *BCL-2* in SW480 and HCT116 cells. (**A**) The 3′-UTR of *BCL-2*-mRNA is a target for miR-21 and the matching sequence is marked in red. The miRNA targets were predicted by Target Scan Human Release 6.2 (http://www.targetscan.org) (accessed on 15 September 2022); (**B**) effect of miR-21 inhibitor on miR-21 expression; (**C**) effect of miR-21 inhibitor on *BCL-2* expression assessed by RT-qPCR. Significant differences were calculated using one-way ANOVA followed by Dunnett’s test (GraphPad Prism 8). Statistical comparisons between cells only vs. miR-21 inhibitor (++++ *p* < 0.0001) and control miRNA inhibitor vs. miR-21 inhibitor (**** *p* < 0.0001). Error bars represent the SEM for six replicates and two independent cultures.

**Figure 10:**
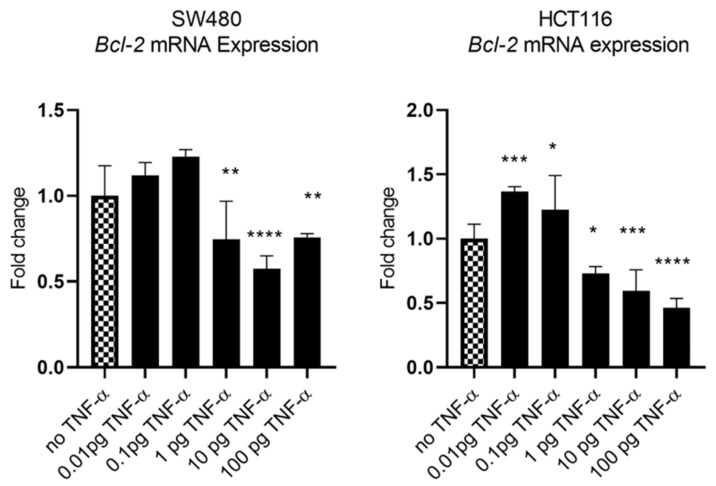
*BCL-2* mRNA expression in SW480 and HCT116 cells treated with TNF-α for 24 h. *BCL-2* mRNA expression was measured by RT-qPCR. Data were normalised to expression of *GAPDH* and are shown relative to control (no TNF-α). Statistically significant differences were calculated using one-way ANOVA with Dunnett’s test (GraphPad Prism 8, * *p* < 0.05, ** *p* < 0.01, *** *p* < 0.001 **** *p* < 0.0001). Error bars represent the SEM for six replicates and two independent cultures.

In our previous study we showed that TNF-α treatment activated the JNK signalling pathway [41]. A downstream target of JNK signalling is the anti-apoptotic protein BCL-2 and, coincidentally, the mRNA of *BCL-2* is a predicted target for miR-21 with complementarity at the 3’ UTR (Figure 9A) (Targetscan; [46]). We, therefore, investigated the effect of TNF-α treatment on *BCL-2* mRNA expression. TNF-α induced a biphasic response on *BCL-2* mRNA expression in both cell lines. At low doses of TNF-α (≤0.1 pg/mL), *BCL-2* was elevated in both cell lines, but this was statistically significant only in HCT116. At a higher dose of TNF-α (≥1 pg/mL), *BCL-2* expression was significantly reduced (Figure 10). We then transfected cells with a miR-21 inhibitor to knockdown miR-21 expression and found that *BCL-2* mRNA expression level was significantly increased in both cell lines (Figure 9), supporting the role of miR-21 in controlling the expression of *BCL-2* mRNA. 

### 3.5. TNF-α Alters VEGF-A Expression, but Not MMP2

**Figure 11:**
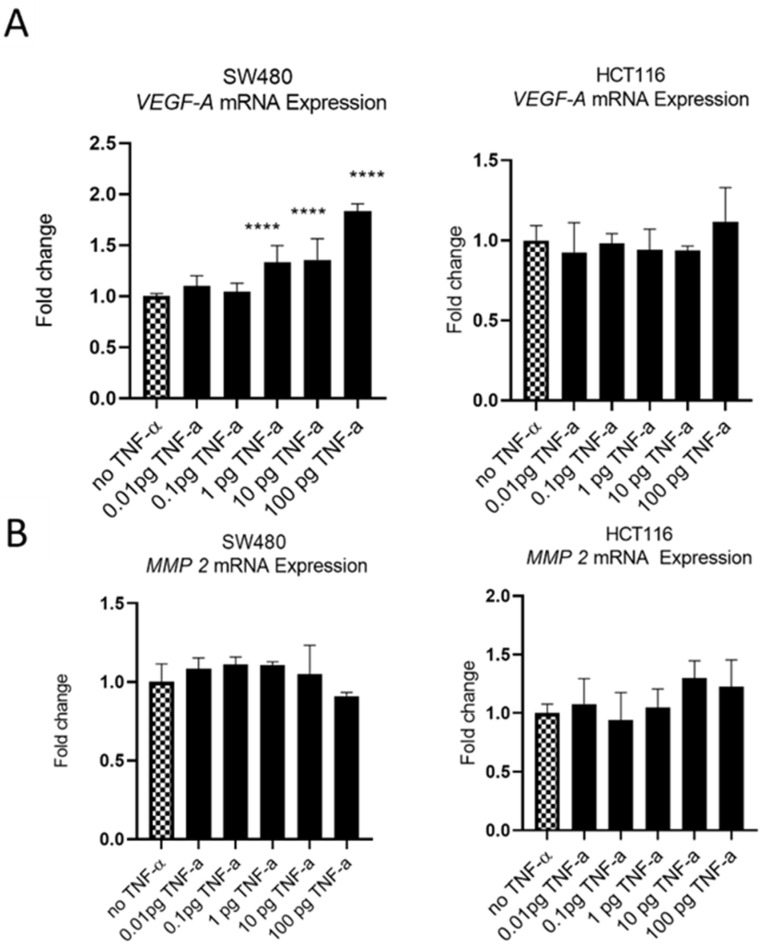
*VEGF-A* (panel (**A**)) and *MMP 2* (panel (**B**)) mRNA expression in response to TNF-α treatment in SW480 and HCT116 cells. Cells were treated with a dose range of TNF-α (0.01–100 pg/mL). Data are expressed as a fold change compared to vehicle control (no TNF-α). Significant difference was calculated using one-way ANOVA followed by Dunnett’s test (GraphPad Prism 8, ****p < 0.0001). Error bars represent the SEM for six replicates and two independent cultures.

The oncoprotein vascular endothelial growth factor A (*VEGF-A*) is pro-metastatic and is implicated in cell migration and invasion. Involved in angiogenesis, elevated levels of *VEGF-A* are a marker of metastasis and tends to be expressed at higher levels in cells with a more aggressive metastatic phenotype [47]. We treatedSW480 cells with TNF-αand found induced *VEGF-A* mRNA expression, in a dose-dependent manner, with induction being statistically significant at ≥1 pg/mL of TNF-α. In contrast, the treatment of HCT116 cells with TNF-α failed to affect the expression of *VEGF-A* at any of the doses employed (Figure 11A). 

Matrix metalloproteinase 2 (MMP2) is another protein that is involved in progressive metastatic transformation of cancer cells, where its protease activity is involved in the digestion of basement membrane proteins, thereby facilitating cellular movement from the primary site [48]. Elevated expression of MMP family proteins is, therefore, characteristic of metastatic cells. However, treatment of SW480 and HCT116 cells with TNF-a failed to alter the mRNA expression of *MMP2* in either cell line (Figure 11B). 

**Figure 12:**
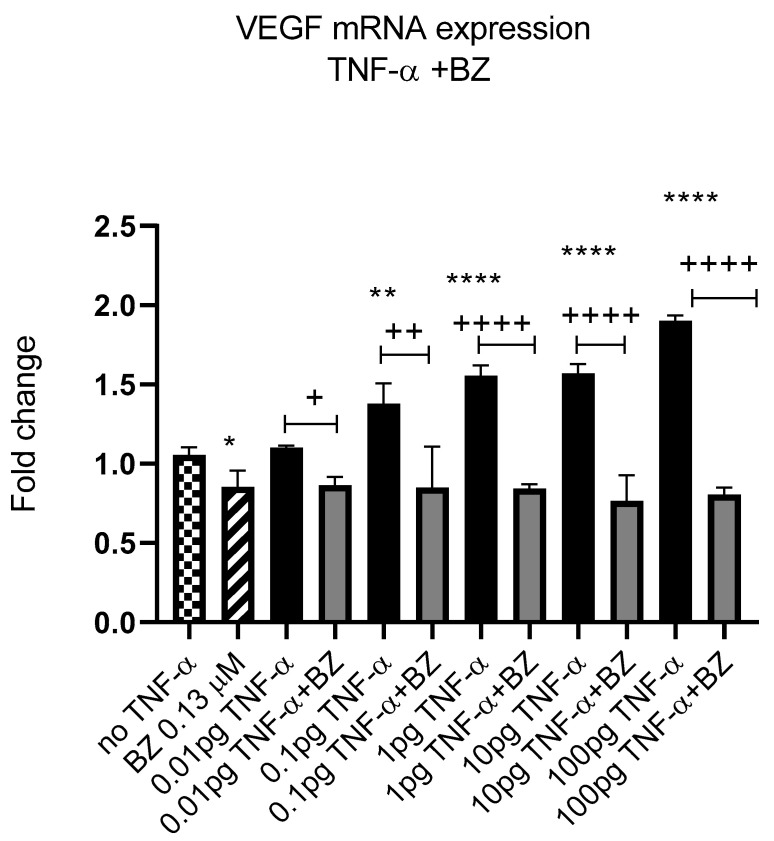
NF-kB involvement in TNF-α-induced VEGF in SW480. Cells were treated with 0.13 μM of Bortezomib (BZ) with TNF-α dose range of (0.01–100 pg/mL) for 24 h. Significant differences are shown for comparisons between inhibitor treated cells vs. untreated cells (+ *p* < 0.05, ++ *p* < 0.01, ++++ *p* < 0.0001) and compared with vehicle control (no TNF-α) (* *p* < 0.05, ** *p* < 0.01, **** *p* < 0.0001). Significance was assessed using one-way ANOVA (GraphPad Prism 8). Data are presented as a mean and error bars represent the SEM for six replicates and two independent cultures.

Constitutively active NF-kB is associated with aggressive features of tumour cells [9,10,11]. We previously reported that TNF-α activated the NF-kB pathway in SW480 cells [41]; therefore, we investigated the effect of NF-kB inhibition on *VEGF-A* expression in SW480 cells. Co-treatment of cells with TNF-α and NF-kB inhibitor bortezomib (BZ) decreased the expression of *VEGF-A* back to control levels compared to TNF-α treatment alone (Figure 12), supporting a role for NF-kB in regulating the expression of *VEGF-A* in CRC cells. 

## 4. Discussion

Metastatic transformation of cells is commonly associated with the presence of inflammatory mediators, such as TNF-α in the TME [49]. Whilst the inflammatory effects of these mediators are well studied and understood, the relationship between TNF-α and the metastatic transformation of cells within a TME is not yet clear. Metastatic transformation is a multifactorial process and the mechanisms involved in TNF-α-mediated phenotypic changes are complex. We have previously reported that miRs are present in the TME and are involved in signalling between cells [31] and, therefore, we hypothesised that inflammatory cytokines, such as TNF-α, may promote the metastatic phenotype via miRs. MiRNAs regulate gene expression by binding to their target mRNA leading to mRNA instability and degradation and stalling of translation; many miRs are dysregulated in the TME [50]. Therefore, understanding the role of miRs in TME is crucial. Pertinent to this, the oncogenic miR-21 has been found to be upregulated in tumours and is strongly associated with TNF-α expression and tumour progression and is a potential key mediator of metastatic potential [25,27,42] and also, therefore, a strong candidate as a TNF-α responsive mediator.

TNF-α concentrations in excess of 36 pg/mL have been reported in the circulation of patients with CRC [13] and our laboratory has reported TNF-α concentrations ranging from 5 to 25 pg/mL present in the TME of CRC patients [15]. Even at these low physiological concentrations, TNF-α remains a potent biological molecule. To the best of our knowledge, we, for the first time, show that at these physiologically relevant concentrations, the inflammatory mediator TNF-α induced miR-21 expression levels in human colonic epithelial cancer HCT116 and SW480 cells in a dose-dependent manner. Previous studies from our laboratory have shown that agents that affect miRNA expression can result in multiple diverse phenotypic alterations [26,31,51,52,53,54]. MiR-21 is reported to affect cell motility and stimulation of EMT [38,55], including the downregulation of genes such as E-cadherin, required to maintain the epithelial phenotype [55]. Having showed that TNF-α treatment enhanced miR-21 expression in both cell lines, we investigated the potential role of miR-21 in metastatic progression and downstream effects on miR-21 targets. 

TNF-α is known to enhance cell migration and invasion, but the underlying mechanisms are still to be defined [56]. Here, treatment with TNF-α encouraged proliferation in SW480 cells, but failed to do so in HCT116 cells. This differential cellular response is likely to be attributable to the differing metastatic phenotype origins of the two cell types (Dukes stage B versus stage D). Interestingly, both cell types harbour *RAS* mutations; the SW480 cell line carries a mutation at codon 13 and HCT116 includes a mutation at codon 12. It is reported that, in terms of colorectal cancer, *RAS* mutation is a frequent event and carries a poorer prognosis compared to wild type *RAS*, but the influence of mutation at codon 12 versus codon 13 on prognosis is unresolved [57]. Additionally, it should be noted that SW480 carries a mutated p53 gene, whereas HCT116 cells are wild type p53, which is likely to impinge on the overall phenotypic characteristics of the two cell lines. On the other hand, the doubling time of both cell lines under similar conditions is comparable (~20 h); therefore, the differential (yet marginal) proliferative response of the two cell lines to TNF-α treatment is difficult to attribute beyond their phenotypic origins. We reported that the knockdown of miR-21, via transfection of a miR-21 inhibitor, led to a significant decrease in SW480 cell proliferation. Consistent with this, Zhong et al. [58] showed that the upregulation of miR-21 revealed a higher proportion of cells at S phase and significantly promoted cell proliferation in human lung cancer cell lines. In addition, the knockdown of miR-21 expression resulted in cell cycle arrest at G2/M phase and inhibited cell proliferation [58]. Others have confirmed an association between miR-21 expression and upregulation of the MAP-Kinase pathway leading to enhanced proliferation, migration, and inhibition of apoptosis [59]. We also used a wound assay, which assesses both cell proliferation and migration; TNF-α treatment induced wound closure in a dose-dependent manner in both cell lines. We then explored the treatment of cells with TNF-α in a transwell migration assay and showed that TNF-α strongly promoted the migration of both HCT116 and SW480 cells. These results are in keeping with studies reporting the promigratory activities of TNF-α in bladder, cervical, and melanoma cancer cells [60,61]. Furthermore, the oncomir miR-21 has been shown to promote cell migration and proliferation in breast cancer cells [38]. Consistent with this latter report, we showed that the knockdown of miR-21 prevented cell migration in SW480 and HCT116 cells, thereby linking TNF-α induction of miR-21 with promotion of cell migration. Interestingly, TNF-α treatment of both SW480 and HCT116 failed to induce a response in our in vitro invasion assay. 

Having established that TNF-α treatment influenced cell proliferation (SW480 cells) and migration (SW480 and HCT116 cells) and that proliferation and migration could be inhibited with a miR-21 inhibitor, we considered the possibility that other proteins strongly associated with metastatic progression may also be affected. Another key process in metastatic progression is EMT. Of note here is that E-cadherin, a biomarker for the epithelial phenotype, was reported to be inversely correlated with miR-21 expression in cervical cancer cells [62]. We show here that the treatment of colon cancer epithelial cells with TNF-α decreased the expression of E-cadherin in SW480 cells in a dose-dependent manner. In contrast, in HCT116 cells, a very low dose TNF-α (0.01 pg/mL) induced E-cadherin expression, but doses ≥1 pg/mL significantly reduced expression in a dose-dependent manner. We also observed that the knockdown of miR-21 increased the expression of E-cadherin. This is consistent with reports from others that miR-21-deficient breast cancer cells exhibited a diminished N-cadherin, snail, and vimentin expression profile [38], supporting the notion that miR-21 plays a mechanistic role in EMT progression. 

Additional characteristics of the metastatic phenotype include sustained proliferation, evasion of apoptosis, and resistance to DNA damage and chromosomal instability. Alotaibi et al. showed that TNF-α treatment enhanced DNA damage and chromosomal instability via activation of the c-Jun N-terminal Kinase (JNK) signalling pathway in colonic epithelial cancer cells [41]. Co-incidentally, a downstream target of JNK signalling is the anti-apoptotic protein B-cell lymphoma 2 (BCL-2) [46]. *BCL-2* mRNA is a predicted and experimentally validated target for miR-21 in several cancers, including breast, lung, and glioblastoma [39,46]. We report here that TNF-α treatment downregulated the expression of the miR-21 target *BCL-2* in both SW480 and HCT116. Conversely, the knockdown of miR-21 via the transfection of the miR-21 inhibitor elevated the expression of *BCL-2*. Thus, our results are consistent with our previous report [41], in which TNF-α enhanced DNA damage via the activation of the JNK pathway, and we extend this finding to the JNK downstream target BCL-2, the inhibition of which, by TNF-α-induced miR-21, might be expected to promote mitochondrial apoptosis dysfunction.

The ability of cancer cells to migrate and invade adjacent normal tissues is an important step in the development of the metastatic phenotype. An influential family of proteins involved in cell migration and invasion is the matrix metalloproteinases. For example, matrix metalloproteinase 2 (MMP2) is involved in the progressive metastatic transformation of cancer cells [48], including colorectal cancers [63]. The protease activity of the MMP family digests basement membrane proteins, thereby facilitating cellular progression from the primary site [48]. However, the treatment of SW480 and HCT116 cells with TNF-α failed to alter *MMP2* expression in either cell line, consistent with the lack of activity of both cell lines in the invasion assay.

VEGF-A is involved in angiogenesis and cell migration and is known to be upregulated in most solid tumours, including CRC [64]. VEGF-A can be secreted by numerous cell types, including epithelial-derived cancer cells, and a high expression of VEGF proteins is a poor prognostic marker [64]. Here, we report that *VEGF-A* is upregulated in SW480 cells treated with TNF-α in a dose-dependent manner, but not in HCT116 cells. The reasons for this differential expression of *VEGF-A* in the two cell lines in response to TNF-α is not obvious from our data, but is again likely to be attributable to the differing phenotype origins of the two cell types (Dukes stage B versus stage D). The current data also showed that inhibition of NF-kB with bortezomib reduced the expression of *VEGF-A*, in line with similar findings in macrophage cells [65] and glioblastoma [66]. 

## 5. Conclusions

In summary, our findings are in agreement with reports of the strong correlation between inflammation and tumour development [8,9,10,11,26] and extends our understanding of these associations. Collectively, our data confirm a role for physiological levels of TNF-α present in the TME in driving the metastatic development of colorectal cancer epithelial cells, and offers mechanistic support for a role of TNF-α-mediated induction of miR-21 in facilitating the progression to the metastatic phenotype. We suggest that a confirmation of these TNF-α-mediated events in appropriate translational studies would support the strategy that targeting miR-21 in cancers such as CRC could offer novel therapeutic opportunities.

## Data Availability

The data presented in this study are available on request from the corresponding author.
